# Application of Multiple Deep Learning Architectures for Emotion Classification Based on Facial Expressions †

**DOI:** 10.3390/s25051478

**Published:** 2025-02-27

**Authors:** Cheng Qian, João Alexandre Lobo Marques, Auzuir Ripardo de Alexandria, Simon James Fong

**Affiliations:** 1 Institute of Data Engineering and Science, University of Saint Joseph, Macau SAR, China; qian.cheng@usj.edu.mo; 2 Laboratory of Applied Neurosciences, University of Saint Joseph, Macau SAR, China; alexandre.lobo@usj.edu.mo; 3 Federal Institute of Education, Science and Technology of Ceará, Fortaleza 60040-215, CE, Brazil; 4 Faculty of Science and Technology, University of Macau, Macau SAR, China; ccfong@um.edu.mo

**Keywords:** facial expression recognition, deep learning, artificial intelligence, model performance evaluation, FER2013 dataset

## Abstract

Facial expression recognition (FER) is essential for discerning human emotions and is applied extensively in big data analytics, healthcare, security, and user experience enhancement. This study presents a comprehensive evaluation of ten state-of-the-art deep learning models—VGG16, VGG19, ResNet50, ResNet101, DenseNet, GoogLeNet V1, MobileNet V1, EfficientNet V2, ShuffleNet V2, and RepVGG—on the task of facial expression recognition using the FER2013 dataset. Key performance metrics, including test accuracy, training time, and weight file size, were analyzed to assess the learning efficiency, generalization capabilities, and architectural innovations of each model. EfficientNet V2 and ResNet50 emerged as top performers, achieving high accuracy and stable convergence using compound scaling and residual connections, enabling them to capture complex emotional features with minimal overfitting. DenseNet, GoogLeNet V1, and RepVGG also demonstrated strong performance, leveraging dense connectivity, inception modules, and re-parameterization techniques, though they exhibited slower initial convergence. In contrast, lightweight models such as MobileNet V1 and ShuffleNet V2, while excelling in computational efficiency, faced limitations in accuracy, particularly in challenging emotion categories like “fear” and “disgust”. The results highlight the critical trade-offs between computational efficiency and predictive accuracy, emphasizing the importance of selecting appropriate architecture based on application-specific requirements. This research contributes to ongoing advancements in deep learning, particularly in domains such as facial expression recognition, where capturing subtle and complex patterns is essential for high-performance outcomes.

## 1. Introduction

Emotions represent physiological states intrinsic to the nervous system, significantly influencing cognitive processes and decision-making within everyday contexts [1]. Facial behavior emerges as a pivotal cue for deciphering human emotions and intentions, serving as a primary modality for interpersonal communication [2,3]. The psychologist Mehrabian proposed that human communication predominantly relies on non-verbal cues, with facial expressions conveying 55% of the information, voice or sounds conveying 38%, and spoken language itself accounting for a mere 7% [4,5]. There are seven basic, recognizable facial emotions that can be understood across cultures and languages even without translation: happiness, sadness, fear, surprise, disgust, and neutral [6]. Computer science and artificial intelligence are advancing rapidly in recent years. Recent strides in computer vision and artificial intelligence illustrate the criticality of emotion analysis systems predicated on facial expression recognition. Such systems find application across diverse domains, including education [7,8,9], computer science [10,11,12,13], big data analytics [14], the Internet of Things [15,16,17], and healthcare. While human interpretation of facial expressions is intuitive, the task poses considerable challenges for machines. Human facial expressions exhibit intricate variations encompassing both macroscopic and microscopic dynamics. Moreover, machines confront obstacles in discerning facial emotions amidst fluctuating environmental conditions, background noise, illumination, and other factors [18]. Despite the potential of different sensory modalities, such as touch and auditory cues, visual analysis remains paramount in assessing emotional states in emotion detection.

In contemporary contexts, facial expression recognition (FER) is increasingly utilized within big data analytics. This technology possesses the capability to yield profound insights into customer behavior. By employing FER, enterprises can effectively observe real-time customer emotions, refine marketing strategies, and improve customer satisfaction. Concurrently, within the domain of user experience (UX), leveraging FER enables interactive interfaces that respond to users’ facial expressions, thereby enhancing the overall user experience, and through extensive big data analysis, underlying patterns, and user preferences can be discerned, driving iterative design improvements. FER also plays a vital role in real-time individual identification and emotion assessment, enhancing security measures. In contexts such as public safety and surveillance, FER systems exhibit proactive capabilities in responding to potential threats and critical events. Educational institutions similarly benefit from FER by gauging students’ engagement and emotional responses, thereby informing more tailored pedagogical strategies. Amidst these benefits, it is imperative to address pertinent ethical concerns, including privacy and consent issues inherent in big data applications. Ensuring robust data security and safeguarding user privacy are paramount in navigating these challenges effectively. Numerous researchers have investigated the application of facial expression recognition within big data. For instance, Xia and Ding [19] delved into the fusion of facial micro-expression recognition with core big data technologies. Deng [20] introduced an enhanced facial detection and recognition methodology leveraging big data techniques; this approach utilized dynamic sequence models for feature extraction, integrating personalized learning alongside optical flow technology to advance dynamic sequence recognition. In the context of extensive virtual data courses, Lu and Ji [21] proposed a method for facial expression extraction based on feature texture mapping. They combined a time threshold processing mechanism into their filtering algorithm to achieve real-time tracking of facial feature points.

In addition to evaluating the general performance of deep learning models for facial expression recognition (FER), this study also considers the practical deployment of these models in Internet of Things (IoT) devices, which typically have limited computational resources. As such, the algorithms selected for this study are primarily Convolutional Neural Networks (CNNs), which have been widely recognized for their computational efficiency and generalization capabilities in constrained environments. While non-CNN-based approaches, such as Vision Transformers (ViTs), have shown promising results in various visual tasks, their computational requirements are often higher, making them less suitable for real-time deployment in IoT systems. Therefore, this study focuses on CNN-based models, which balance accuracy and computational efficiency, making them well-suited for IoT applications.

Nevertheless, gaps exist in the comparative analysis of various deep-learning models for facial expression recognition. Numerous open-source databases exist for this purpose, necessitating further investigation into model performance using consistent datasets. The main objective of this work is to evaluate and compare the performance of ten deep learning models (VGG16, VGG19, ResNet50, ResNet101, GoogLeNet V1, DenseNet, RepVGG, MobileNet V1, ShuffleNet V2, and EfficientNet V2) on the Facial Expression Recognition 2013 (FER2013) database. The proposed approach contributes with empirical insights by assessing their respective metrics within the same dataset.

This article is a revised and expanded version of the conference paper entitled “Analysis of Deep Learning Algorithms for Emotion Classification Based on Facial Expression Recognition”, which was presented at the 8th International Conference on Big Data and Internet of Things (Macau, China, September 2024) [22]. In the original conference paper, we conducted a comparative analysis of the performance of algorithms including VGG16, DenseNet, ResNet50, and GoogLeNet on the FER2013 dataset, evaluating a range of metrics. In this enhanced journal article, we have extended the analysis to include ten algorithms: VGG16, VGG19, ResNet50, ResNet101, DenseNet, GoogLeNet V1, MobileNet V1, EfficientNet V2, ShuffleNet V2, and RepVGG. Additionally, we provide a more comprehensive analysis, including a detailed comparison of the performance at different training stages, such as model convergence speed and the analysis of individual algorithm modules.

## 2. Related Works

Emotional states can swiftly propagate among individuals through facial expressions. Within computer vision and artificial intelligence, the study of emotion analysis via facial expression recognition holds significant implications for emotion computing. Recent years have witnessed heightened scholarly attention toward this area, prompting researchers to propose diverse methodologies. For instance, Sathya et al. [23] utilized support vector machines (SVM) with radial basis function (RBF) kernels for emotion classification, emphasizing preprocessing, face recognition, and feature extraction. Siam et al. [24] introduced an approach wherein generated key points are encoded through a sequence of meticulously designed mesh generators and angular encoding modules. Additionally, various decomposed feature techniques have been explored, employing algorithms such as SVM, K-nearest neighbors (KNN), naive Bayes (NB), logistic regression (LR), and random forest (RF). Tiwari et al. [25] conducted a comparative analysis of SVM and NB algorithms, specifically in facial expression recognition, highlighting the efficacy of mouth region feature extraction in enhancing recognition accuracy. Furthermore, Hemmatiyan et al. [26] evaluated diverse methods for feature extraction and classification, encompassing Histogram of Oriented Gradient (HOG), Adaptive Boosting (AdaBoost), and Logistic Regression (LR), underscoring the holistic importance of facial image components for accurate expression recognition.

Facial expression recognition necessitates the application of machine learning or deep learning methodologies. Traditional machine learning techniques such as Support Vector Machines (SVM) [27], Decision Trees (DT), naive Bayes (NBM), and Random Forests (RF) [28] were previously employed; additionally, early facial expression recognition relied on methods like Local Binary Pattern (LBP) and Histogram of Oriented Gradient (HOG) [29]. These traditional approaches required significant manual effort for feature engineering and extensive data annotation. They also encountered challenges with variations in illumination, partial face occlusions, and other conditions. In contrast, deep learning has gained prominence among researchers. The breakthrough of AlexNet [30] in the 2012 ImageNet competition, surpassing competitors by a substantial margin of 10.9%, marked a turning point for CNNs, catalyzing their widespread adoption of computer vision and advancing the field significantly. Since then, diverse CNN architectures like VGGNet [31], ResNet [32], and Inception [33] models have emerged, each introducing distinctive innovations to artificial intelligence and computer vision. Convolutional Neural Networks (CNN) emulate the human visual nervous system, autonomously extracting and learning features directly from data, thereby circumventing the labor-intensive feature engineering inherent in traditional supervised learning methods.

With artificial intelligence and deep learning advancements, researchers have increasingly utilized deep learning technologies to develop Facial Expression Recognition (FER) systems. For instance, Dwijayanti et al. [34] conducted a comparative study using AlexNet and VGG16 models to determine optimal implementation in humanoid robots. Bhargavi et al. [35] integrated three transfer learning architectures, namely VGG16, MobileNet, and ResNet50, into a treatment robot aimed at early detection of autism spectrum disorder (ASD) individuals and formulation of tailored treatment strategies, noting that the VGG16 architecture achieved the highest identification accuracy at 97.66%. Han et al. [36] proposed a tri-structure network model based on MobileNet V1, trained with a new multi-branch loss function. Faiyaz et al. [37] utilized ShuffleNet V2 for facial emotion analysis, applying it to driver emotion recognition. Xu et al. [38] developed a dual-channel model combining RepVGG and MobileNet V2, replacing the global pooling output with capsule network pose vectors. This approach preserves the spatial relationships of salient features, enhancing classification performance and enabling lightweight, real-time facial expression recognition. Gupta et al. [39] proposed a deep learning-based approach for calculating an online learner engagement index, evaluating and comparing it with models such as VGG19 and InceptionV3 to develop a real-time user engagement detection classification model. Agrawal et al. [40] explored adjustments to the complexity and diversity of pre-trained models, including VGG16, VGG19, AlexNet, and ResNet50, affirming that transfer learning methods facilitate effective prediction of human emotions.

In recent years, Vision Transformer (ViT) has emerged as a promising approach in the field of computer vision, including applications in facial expression recognition [41]. ViT leverages self-attention mechanisms, originally designed for natural language processing tasks, to capture long-range dependencies in image data [42]. Unlike traditional convolutional neural networks (CNNs), which focus on local spatial patterns, ViT processes the image as a sequence of non-overlapping patches and applies attention across these patches to extract relevant features [43].

Several studies have explored the potential of ViT for facial expression recognition. For example, Kim et al. [44] introduced Squeeze ViT, which enhances FER by combining global and local features to overcome the limitations of ViT in capturing subtle facial expressions. Their method outperformed previous models on both controlled and wild FER datasets. Liu et al. [45] proposed the Patch Attention Convolutional Vision Transformer (PACVT) to handle occlusion in FER by using a CNN backbone and ViT to focus on discriminative facial patches. PACVT achieved superior performance on occluded FER datasets. Xue et al. [46] proposed TransFER, a transformer-based method for FER that uses Attentive Patch Pooling (APP) and Attentive Token Pooling (ATP) to focus on the most discriminative features while reducing computational cost. Their approach outperformed state-of-the-art methods on multiple in-the-wild FER datasets. Chaudhari et al. [47] applied ViT for FER, using ResNet-18 and transformers for efficient feature extraction and classification, showing strong performance on hybrid FER datasets. Huang et al. [48] proposed a CNN-based FER framework with two attention mechanisms: grid-wise for local features and transformer attention for global features, achieving state-of-the-art results on multiple FER datasets. Thus, Vision Transformer models represent an important advancement in FER systems, providing a powerful alternative to conventional methods like VGG16, ResNet, and MobileNet. As FER research progresses, the application of ViT is expected to further enhance the accuracy and robustness of facial emotion detection, paving the way for more sophisticated and adaptable models.

In conclusion, the studies outlined above, from conventional machine learning algorithms to advanced deep learning methodologies, are dedicated to enhancing the efficacy of facial expression recognition models. By refining and adapting established models, these studies thoroughly contemplate all facets of model design and the practical implications of the proposed frameworks in facial expression recognition applications. The evaluation of classical model accuracy in Facial Expression Recognition (FER) holds paramount significance for the progression of FER systems. These studies collectively establish a robust groundwork for facial expression recognition based on deep learning techniques.

## 3. Proposed Methodology

### 3.1. Dataset Description

Facial Expression Recognition (FER) has been a prominent research area, with many publicly available datasets contributing to the development and evaluation of models in this field. These datasets offer a variety of emotional expressions and contextual variations, enabling comprehensive training and testing of deep learning models. In this study, we selected the Facial Expression Recognition 2013 (FER2013) dataset [49] as our training and validation dataset due to its widespread use in the literature and large-scale nature, making it particularly suitable for deep learning applications. The FER2013 dataset was initially showcased in a Kaggle competition and contains 35,887 grayscale images, each 48 × 48 pixels in size, categorized into seven emotional expressions: anger, happiness, fear, disgust, sadness, surprise, and neutral. These categories are widely accepted as the basic facial expressions in emotional psychology research. Table 1 provides additional details about the dataset. Given the widespread use of FER2013, it allows for meaningful comparisons with other studies and ensures the reproducibility of results. The large scale of the dataset, coupled with its academic prominence [50,51,52], makes it an ideal choice for training and validating deep learning models. Furthermore, as one of the largest publicly available FER datasets, it offers a balance between diversity and consistency, supporting the model’s generalization across various facial expressions.

**Figure 1:**
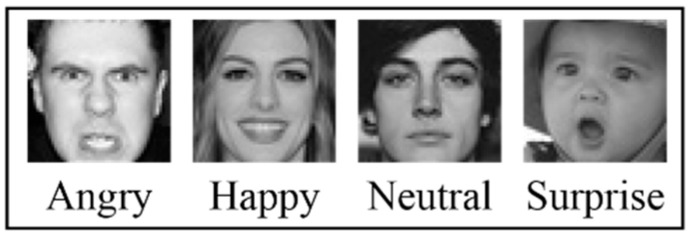
Standard Samples of the FER2013 Database.

**Figure 2:**
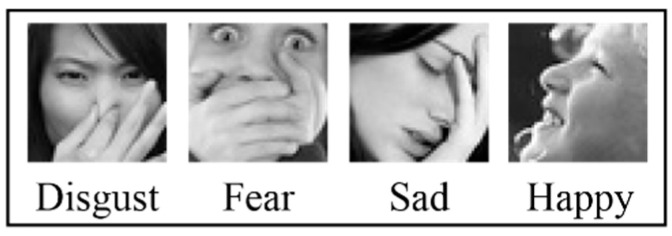
Non-standard Samples of the FER2013 Database.

Several limitations and challenges are evident in this dataset, including unbalanced data distribution, with a mere 547 disgust images, representing just 1.5% of the total. Furthermore, factors such as facial occlusion or illumination and the potential for mislabeling introduce additional complexities. The visual representations provided in Figure 1 and Figure 2 showcase examples of standard and non-standard image samples, highlighting discernible differences between the two categories. Notably, situations arise where facial emotions are challenging to detect, particularly in instances of facial occlusion. Moreover, occasional mislabeling of individual images occurred; however, to ensure continuity with prior research, these labels remained unaltered in this study.

### 3.2. Experiment Setup and Training Parameters

In this study, the following parameters were used for model training: a learning rate of 0.001, a batch size of 64, the Adam optimizer, and 100 training epochs. These parameter settings were derived through a combination of previous research and empirical experimentation to strike a balance between training efficiency, model performance, and resource consumption.

#### 3.2.1. Learning Rate

The learning rate controls the stride during the optimization process. Based on previous research and empirical validation, a value of 0.001 was selected because it provides an effective balance between stable convergence and fast learning. A higher learning rate may prevent the model from reaching the optimal point, while a lower learning rate can result in slower convergence. This selection is consistent with commonly used parameter settings for similar classification tasks in deep learning [53], where the Adam optimizer typically performs well at a learning rate of 0.001.

#### 3.2.2. Batch Size

In this study, a batch size of 64 was chosen to balance computational efficiency and memory utilization. In deep learning, batch size directly influences the dynamic process of training. Smaller batch sizes generally allow the model to converge faster, but they can introduce higher variance in the gradients, while larger batch sizes can reduce variance but may require more memory and computational power. Choosing a batch size of 64 ensures stable updates to model parameters without overburdening the system, particularly when training on large datasets such as FER2013.

#### 3.2.3. Training Epochs

Based on the early findings of this study, the training of this study was set at 100 epochs. Previous research [22] has shown that when training epochs are set to 200, all models tend to stabilize between 50 and 75 epochs. This phenomenon suggests that for traditional CNN models in facial expression classification tasks, model performance stabilizes after approximately 50 to 75 epochs, with little to no further improvement thereafter. Therefore, this study trained the model for 100 epochs, ensuring convergence while avoiding unnecessary computation after the model’s performance stabilized.

#### 3.2.4. Data Augmentation

This study employed random horizontal flipping and random erasing as data augmentation techniques to enhance the model’s generalization ability and reduce the risk of overfitting. Random horizontal flipping helps simulate different viewpoints, improving the model’s robustness. The random erasing technique is applied to random regions of the entire image, with the erased area covering between 0.02 and 0.1 of the image, helping the model learn and reducing its sensitivity to facial occlusions and noise. These data augmentation strategies are widely used in FER tasks and have been proven to improve the robustness of the model in real-world applications.

These were the parameter settings used in this study to ensure effective model training while achieving stable model performance. Table 2 summarizes these parameters, which have been validated through extensive experimentation and align with best practices in deep learning for image classification tasks, especially in the context of facial expression recognition.

### 3.3. IoT Constraints and the Choice of Models

While Vision Transformers (ViT) have demonstrated impressive performance in a variety of computer vision tasks, their application in Facial Expression Recognition (FER) for Internet of Things (IoT) environments presents several challenges that make alternative deep learning models more suitable. IoT systems are often characterized by resource constraints, such as limited computational power, memory, and energy availability. ViTs, while effective in capturing long-range dependencies through self-attention mechanisms, require substantial computational resources and memory bandwidth, making them less optimal for deployment in resource-constrained IoT environments.

In contrast, the models evaluated in this study—such as VGG16, ResNet50, and EfficientNet V2—offer a more efficient balance between accuracy and computational efficiency. These models, particularly lightweight architectures like MobileNet V1 and ShuffleNet V2, are designed to optimize performance while minimizing resource usage, making them well-suited for real-time facial expression recognition in IoT applications where low latency and low power consumption are critical. Thus, while ViTs excel in capturing complex patterns, the trade-off in computational cost makes them less viable for deployment in IoT systems where energy efficiency and processing speed are paramount.

### 3.4. Deep Learning Algorithms

In computer vision and deep learning, convolutional neural networks (CNNs) are pivotal tools for image analysis and pattern recognition, with significant influence and promise. The models evaluated in this study are predominantly based on Convolutional Neural Networks (CNNs), owing to their proven efficiency in image recognition tasks coupled with relatively low computational overhead. This makes CNNs particularly suitable for deployment in resource-limited environments such as Internet of Things (IoT) devices. Given the computational constraints typical of many IoT systems, architectures such as EfficientNet, ResNet, and MobileNet have been selected for their optimal trade-off between accuracy and computational efficiency. However, with the advent of Vision Transformers (ViTs) and other non-CNN architectures that have demonstrated superior performance across a range of benchmarks, future research will focus on adapting these advanced models for IoT applications, where computational limitations continue to pose significant challenges.

In recent years, several influential CNN architectures, such as VGG16, VGG19, ResNet50, DenseNet, GoogLeNet V1, and EfficientNet V2, etc., have garnered considerable acclaim for their distinctive design principles and outstanding performance. VGG16 and VGG19 are notable for their deep and uncomplicated convolutional layer setup. ResNet50 and ResNet101 resolve gradient vanishing during deep network training with residual connections. DenseNet achieves parameter reduction by employing dense connectivity patterns. GoogLeNet V1 enhances feature extraction efficiency using parallel Inception modules. MobileNet V1 reduces parameters and computation with depthwise separable convolutions for mobile efficiency. EfficientNet V2 enhances training and inference speed using Fused-MBConv blocks and progressive learning strategies. ShuffleNet V2 improves efficiency with channel split and shuffle techniques for lightweight models. RepVGG combines multi-branch training structures with single-path reparameterization for fast inference. The specific model parameters are shown in Table 3. This research aims to examine and contrast these well-established CNN models concerning their architecture, performance, and application in the facial expression recognition area.

#### 3.4.1. VGG16 Network Structure

The VGG16 network model consists of 16 layers, comprising 13 convolutional layers for feature extraction and three fully connected layers for classification. The Rectified Linear Unit (ReLU) is the activation function after each convolutional and linear layer, except for the final one. This choice of activation function helps mitigate the issue of vanishing gradients. Furthermore, each convolutional layer is followed by batch normalization, which normalizes the input to the layer, aiding in faster training and enhanced performance.

Despite its relatively simple architecture, VGG16 has a significant drawback in terms of computational complexity and model size [54]. The large number of parameters, especially in the fully connected layers, results in considerable memory and computational requirements. Additionally, the lack of efficient use of computational resources can make VGG16 unsuitable for deployment in resource-constrained environments, such as mobile devices or embedded systems, without further optimization.

#### 3.4.2. VGG19 Network Structure

The VGG19 network model consists of 19 layers, including 16 convolutional layers used for feature extraction and three fully connected layers for classification. Similar to its predecessor, VGG16, the ReLU is applied as the activation function following each convolutional and fully connected layer, except for the final output layer. The utilization of ReLU addresses the vanishing gradient problem, ensuring stable gradient propagation during training. Additionally, batch normalization is often employed after each convolutional layer, which helps to normalize the input distributions, leading to faster convergence and improved overall performance of the model.

Similar to VGG16, the VGG19 model also suffers from issues related to high computational cost and large memory requirements. The deeper architecture compared to VGG16 increases the number of parameters, which can lead to longer training times and reduced efficiency. Furthermore, the absence of advanced architectural innovations (e.g., residual connections or efficient convolution techniques) limits the model’s scalability and practical application in real-time or resource-limited scenarios.

#### 3.4.3. Resnet50 Network Structure

ResNet50 is a popular convolutional neural network distinguished by its residual learning framework. The model features multiple convolutional layers and utilizes the ReLU activation function to introduce nonlinearity after each batch normalization layer. A key aspect of ResNet50 is its use of BasicBlock residual blocks, each comprising two convolutional layers and a shortcut connection that skips one or more layers. This shortcut connection aids in training extremely deep networks. ResNet50’s deep architecture and residual connections allow for the creation of intense neural networks that exhibit improved performance and training stability.

Although ResNet50 alleviates the vanishing gradient problem with residual blocks, it still faces challenges related to model complexity and training times, particularly in large-scale datasets. The depth of the network increases the risk of overfitting, especially when training on smaller datasets, and its computational requirements can be significant.

#### 3.4.4. Resnet101 Network Structure

The ResNet101 architecture, consisting of 101 layers, utilizes residual blocks with skip connections (identity mappings) to effectively mitigate the vanishing gradient problem, facilitating the training of deep networks. These skip connections allow the network to learn residual functions, simplifying optimization. The ReLU activation function is applied after each convolutional layer, except the output layer, to ensure non-linearity. Batch normalization follows each convolutional layer to normalize input distributions, accelerating convergence and improving model performance.

While the depth of ResNet101 helps capture complex features, the increased number of layers introduces greater computational cost, leading to longer training and inference times. The model also has higher memory demands, making it less suitable for deployment in resource-constrained environments such as mobile devices or embedded systems. Moreover, the model may suffer from vanishing gradients or overfitting on smaller datasets due to its depth.

#### 3.4.5. DenseNet Network Structure

The DenseNet model is a convolutional neural network known for its dense connectivity pattern. It contains several convolutional layers, including DenseLayers and initial convolutional layers. A DenseLayer is the fundamental unit of a DenseBlock, comprising batch normalization, ReLU activation, and two convolutional layers. DenseBlocks are sequences of DenseLayers connected along the channel dimension. In DenseBlocks, each layer receives feature maps from all preceding layers within the block, promoting feature reuse. Additionally, the model uses a compression ratio to reduce the number of feature maps in transition layers between DenseBlocks.

The limitation of DenseNet is high memory consumption due to the dense connections between layers, which result in a large number of feature maps being stored during the forward pass. This can lead to increased computational complexity and longer training times, particularly in large-scale applications.

#### 3.4.6. GoogLeNet V1 Network Structure

The GoogLeNet V1 model is recognized for its deep architecture and innovative modules. It includes an Inception module and an auxiliary classifier, employing average pooling and dropout for regularization, and concludes the classification process with a fully connected layer. The Inception module comprises multiple parallel convolutional branches with varying kernel sizes (1 × 1, 3 × 3, and 5 × 5) and a max pooling layer. The outputs of these branches are concatenated along the channel dimensions. GoogLeNet V1’s key feature is its use of the Inception module, which enables effective feature capture across different spatial scales. This design facilitates the training of deep neural networks, enhancing performance and efficiency.

One of the primary drawbacks of GoogLeNet V1 is its complexity in network design, which involves multiple convolutional branches within the Inception module. While this provides flexibility in feature extraction, it can also make the network prone to overfitting on smaller datasets. Moreover, the model’s reliance on multiple branches can increase training time and computational requirements, making it less efficient in real-time applications where speed is crucial.

#### 3.4.7. MobileNet V1 Network Structure

MobileNet V1 is a lightweight convolutional neural network (CNN) designed for mobile and embedded devices optimized for resource-constrained environments. It introduces Depthwise Separable Convolutions, which divide a standard convolution into two stages: first, a depthwise convolution applies spatial filtering independently to each input channel, followed by a pointwise convolution (1 × 1 convolution) that combines the outputs across channels. This approach significantly reduces both the number of parameters and computational complexity. The model architecture is also highly flexible, allowing adjustments to the network’s width and input resolution to balance trade-offs between accuracy and efficiency based on specific application requirements.

Although MobileNet V1 is efficient, its low accuracy relative to heavier models can be a significant drawback, particularly when high classification performance is required. The trade-off between model size and accuracy may limit its applicability in tasks requiring highly accurate recognition. Additionally, the limited depth of the network can affect its ability to capture complex patterns in challenging image recognition tasks.

#### 3.4.8. EfficientNet V2 Network Structure

EfficientNet V2 represents a significant advancement over its predecessor, with a primary focus on accelerating training times while simultaneously enhancing inference speed. A notable innovation in this version is the implementation of the Fused-MBConv module, which optimizes computational efficiency within the network architecture. Moreover, EfficientNet V2 adopts a progressive learning strategy, including regularization techniques—such as Dropout and RandAugment—that is dynamically adjusted in response to the size of the training images [55]. This adaptive approach facilitates the effective scaling of both model complexity and data augmentation, resulting in substantial improvements in training speed, model accuracy, and overall performance metrics.

Despite its improvements, EfficientNet V2 may still suffer from complexity in tuning and hyperparameter optimization due to its scalable architecture. Fine-tuning the model for different applications may require significant computational resources and time, especially in the context of real-time applications. Additionally, the adaptive approach may not always guarantee optimal performance across diverse tasks or datasets without further refinement.

#### 3.4.9. ShuffleNet V2 Network Structure

ShuffleNet V2 is an efficient neural network designed for high computational performance, accuracy, and lightweight deployment. Its core principle revolves around the innovative rearrangement of channels to facilitate efficient computation. Additionally, the architecture incorporates lightweight modules within its layer design, enabling rapid inference and training on resource-constrained devices [56]. Specifically, ShuffleNet V2 achieves its efficiency through several key techniques, including but not limited to Channel Split operations, adaptive grouped convolutions, multi-scale feature fusion, and channel pruning. These methods collectively contribute to optimizing the network’s performance while maintaining a minimal footprint, making it particularly suitable for mobile and embedded applications.

Although ShuffleNet V2 is optimized for efficiency, its performance may not match that of larger, more complex networks on tasks that require high accuracy. The compact architecture and channel pruning techniques can reduce the model’s ability to capture complex features, limiting its use in scenarios where top-tier performance is essential.

#### 3.4.10. RepVGG Network Structure

RepVGG aimed to optimize the architecture of modern convolutional neural networks to enhance inference efficiency. During the training phase, it employs a multi-branch structure while transitioning to a single-branch structure during inference, thereby reducing memory consumption and improving speed. The multi-branch architecture in the training stage, similar to the residual connections in ResNet, enhances the model’s representational capacity. In the inference phase, RepVGG integrates the multi-branch structure into a single-branch configuration through reparameterization, which not only accelerates inference speed but also decreases memory usage. This approach effectively maintains training performance while significantly enhancing inference efficiency.

While RepVGG enhances inference efficiency, its multi-branch structure during training can still lead to increased computational demands. However, this structure helps improve the model’s representational capacity during training. The key advantage of RepVGG lies in the reparameterization step, which transforms the multi-branch structure into a single-branch structure during inference, significantly reducing computational overhead. This approach simplifies deployment by lowering the memory and computational requirements for inference. However, the reparameterization process may introduce challenges in maintaining model consistency across different deployment environments, particularly if hardware or software frameworks do not support efficient reparameterization.

## 4. Results

### 4.1. Model Evaluation Analysis

In this research, the performance of VGG16, VGG19, ResNet50, ResNet101, DenseNet, GoogLeNet V1, MobileNet V1, EfficientNet V2, ShuffleNet V2, and RepVGG models for facial expression recognition was evaluated. Vital metrics such as training time, model size, test accuracy, and other relevant parameters were analyzed. We analyzed vital metrics such as training time, model size, test accuracy, and other relevant parameters. The output for these metrics is shown in Table 4. The models were trained using the FER2013 training set, comprising 28,709 images categorized into seven classes, with the test set containing 7178 images. Implementation was carried out in Python within the environments of Python 3.11 and PyTorch 2.1.2. Training and validation utilized a 24 GB NVIDIA GeForce 4090 server. The classification cross-entropy was chosen as the loss function concurrently with adopting the Rectified Linear Unit (ReLU) activation function, which is important in non-linear transformation.

#### 4.1.1. Test Accuracy Comparison

Through a comparative analysis of accuracy, the experimental results presented in Table 3 reveal significant differences among various deep learning architectures in the domain of facial expression recognition. EfficientNet V2 achieves the highest accuracy at 68.7%, followed closely by ResNet50 and ResNet101, both at 68.1%. In contrast, GoogLeNet V1 (64.4%) and ShuffleNet V2 (62.3%) exhibit comparatively lower performance.

Based on the characteristics of the different models, this study attributes the superior accuracy of EfficientNet V2 to its foundational principles inherited from EfficientNet V1, particularly the compound scaling method. This unified approach optimally adjusts the model’s depth, width, and resolution to achieve a balance between performance and efficiency. The V2 version refines these parameters further, resulting in a more efficient model. Additionally, EfficientNet V2 employs a progressive learning strategy that begins training on low-resolution images and gradually increases the resolution. This method facilitates faster convergence and helps reduce training time. Conversely, the lower accuracy observed in GoogLeNet V1 and ShuffleNet V2 indicates that, while the inception modules in GoogLeNet are innovative, they rely on a foundational architecture that may not fully exploit deeper feature representations when compared to more contemporary frameworks. This limitation prevents them from capturing the complex patterns and features inherent in facial expression data as effectively as EfficientNet V2. Regarding ShuffleNet V2, its design is primarily oriented towards efficiency for mobile devices, emphasizing lightweight and real-time performance. Although this model combines channel shuffling and depthwise separable convolutions to reduce complexity, it necessitates a compromise in accuracy as a trade-off.

In terms of other models, both ResNet50 and ResNet101 achieved an accuracy of 68.1%, largely benefiting from the use of residual connections. These connections address the common issue of vanishing gradients in deep networks, enabling more effective training of deeper architectures that can learn complex features crucial for capturing subtle variations in facial expressions. Despite the increased depth of ResNet101, the lack of significant accuracy improvement over ResNet50 suggests that beyond a certain threshold, the returns on increasing network depth may diminish or plateau. Compared to advanced algorithms, traditional models such as VGG16 (67.7%) and VGG19 (67.1%), while historically significant and yielding respectable accuracy, fall short of the performance demonstrated by more modern architectures like EfficientNet V2 and ResNet. The substantial parameter counts in the VGG series stem from their deep, fully connected layers, which may be a contributing factor to their relatively lower performance. DenseNet, achieving an accuracy of 67.6%, employs dense connectivity to encourage feature reuse, thus enhancing parameter efficiency throughout the network. While this architectural paradigm offers notable advantages in network design, its performance remains 1.2% lower than the highest accuracy achieved in this study by EfficientNet V2. These differences highlight the imperative for ongoing innovation in model development to attain optimal performance in facial expression recognition tasks.

#### 4.1.2. Training Time Comparison

The evaluation of training time per epoch for various deep learning models in facial expression recognition revealed significant disparities, reflecting the inherent structural differences and computational complexity of each model. Notably, ShuffleNet V2 exhibited the shortest training time, at 20.6 s per epoch (s/epoch), which can be attributed to its design that emphasizes lightweight operations and computational efficiency, such as channel shuffling and depthwise separable convolutions. These methods make it particularly well-suited for real-time applications on resource-constrained devices. RepVGG, with a training time of 21.6 s/epoch, also demonstrated high efficiency. Its simplified architecture allows for rapid convergence and effective training by minimizing the overhead typically associated with more complex models, enabling faster training iterations without a significant impact on accuracy. Compared to ShuffleNet V2, RepVGG achieved a 3.8% higher accuracy while maintaining similar training efficiency. MobileNet V1 achieved a training time of 30.8 s/epoch thanks to its lightweight architecture that employs depthwise separable convolutions to reduce computational load. In contrast, EfficientNet V2 required 46.2 s/epoch, a longer training time due to the increased computational demands imposed by its compound scaling method. However, EfficientNet V2 balances this by delivering improved accuracy and optimizing performance through efficient resource utilization, although it inevitably requires more time compared to lighter models.

ResNet50 and ResNet101 have comparable training times, at 44.4 and 44.7 s/epoch, respectively. Their use of residual connections facilitates more effective training by mitigating the vanishing gradient problem, but the increased depth and parameter count in these models lead to longer training durations compared to simpler architectures. DenseNet, with a training time of 54.3 s/epoch, employs dense connectivity to promote feature reuse across the network. While this design enhances parameter efficiency, the increased complexity results in longer training times compared to simpler models, as reflected in the experimental outcomes. Finally, VGG16 and VGG19 exhibited the longest training times, at 79.8 and 80.6 s per epoch, respectively. Their extensive depth and reliance on fully connected layers contribute to a large number of parameters, demanding significant computational resources. This complexity inherently leads to prolonged training durations, which may hinder rapid iterations during model development.

#### 4.1.3. Weight File Size Impact

The size of the weight files for deep learning models reflects the complexity of the architecture, the number of parameters, and the overall design of the models. In this study, the differences in weight file sizes among the models were striking, with each model exhibiting its unique structural characteristics. VGG16 and VGG19 have the largest weight files, both at 512 MB. This is indicative of the VGG series model’s heavy reliance on depth, fully connected layers, and a large number of parameters, which significantly increases the weight file size. While this design enables the capture of fine-grained features, it results in inefficiencies in storage and computational requirements, rendering these models less suitable for applications where storage or memory is a primary constraint. In contrast, ResNet50 and ResNet101 had weight file sizes of 81.3 MB and 158 MB, respectively. Despite their deeper architecture and the inclusion of numerous layers, residual connections allow for more efficient parameter sharing and utilization. The larger file size of ResNet101, as compared to ResNet50, can be attributed to its increased depth, which introduces additional parameters. DenseNet, with a weight file size of 27.1 MB, benefits from its dense connectivity pattern, which promotes cross-layer parameter reuse, effectively reducing the total number of parameters. Compared to the VGG models, its smaller weight file size highlights the advantage of modern architectural innovations in reducing storage overhead. As for GoogLeNet V1, it has a small weight file size of 39.4 MB, utilizing the Inception module, which includes multiple convolution operations of different sizes within a single layer, allowing for a more compact and efficient design. While its weight file is larger than lightweight models like MobileNet V1 and ShuffleNet V2, GoogLeNet V1 strikes a good balance between model complexity and storage efficiency.

Notably, EfficientNet V2 achieved a balanced trade-off between performance and weight file size, with a file size of 77.8 MB. Its compound scaling method optimizes the network’s depth, width, and resolution in a balanced manner, resulting in a compact weight file size while maintaining high accuracy. This reflects the model’s design philosophy of maximizing performance while minimizing resource usage. ShuffleNet V2, with the smallest weight file size among all models at 1.47 MB, directly demonstrates its focus on lightweight operations, including channel shuffling and depthwise convolutions, which drastically reduce the number of parameters. This extremely small file size makes ShuffleNet V2 highly suitable for devices with limited storage and computational capacity. Additionally, RepVGG, with a weight file size of 10.7 MB, also achieves a balance between architectural simplicity and efficiency. By using a re-parameterization strategy, which simplifies the structure during inference, RepVGG achieves a low-weight file size while maintaining competitive performance levels. Its simplicity and small weight file size make it an attractive option for real-time deployment scenarios where speed and storage efficiency are prioritized.

In conclusion, the observed differences among these models can be attributed to innovations in their network architecture, parameter configurations, and the efficiency of feature extraction and representation. In terms of accuracy, EfficientNet V2’s compound scaling method, the residual connections in the ResNet series models, and DenseNet’s dense connectivity all demonstrate the necessity of advances in modern model architecture and their role in enhancing algorithm performance. Conversely, the limitations of traditional models like the VGG family and GoogLeNet underscore the importance of continuous innovation in deep learning methodologies. Regarding training time, lighter models such as ShuffleNet V2 and RepVGG significantly reduce training durations, whereas more complex architectures like VGG and DenseNet require longer training times, highlighting the trade-off between model efficiency, accuracy, and training speed in facial expression recognition tasks. As for the weight file size, traditional models like VGG16 and VGG19 result in larger weight files due to their deep and parameter-heavy architectures, while more modern models such as ShuffleNet V2 and MobileNet V1 achieve smaller weight files through the use of lightweight convolution techniques. Meanwhile, models like EfficientNet V2 and the ResNet architectures strike a balanced trade-off between complexity, accuracy, and storage efficiency. These distinctions emphasize the critical importance of model design and selection when determining the practical deployment of deep learning algorithms, particularly in environments with limited computational resources.

### 4.2. Expression Recognition Performance Analysis

In this study, we chose to use the confusion matrix as the primary method for evaluating and comparing the performance of the models. The confusion matrix is widely regarded as a critical tool for assessing the performance of classification models, particularly in multi-class tasks like facial expression recognition (FER). It offers a comprehensive view of the model’s ability to correctly classify each category, as well as its tendency to misclassify instances into other categories [56,57].

The confusion matrix presents a detailed analysis of the true positives (TP), false positives (FP), true negatives (TN), and false negatives (FN) for each class, which allows for the calculation of essential metrics such as accuracy, precision, recall, and F1-score [58,59]. These metrics are pivotal in understanding not only the overall accuracy of the model but also its ability to handle class imbalances, which is particularly important in FER tasks where some expressions (e.g., disgust, fear) may have fewer samples compared to others. By providing insight into the number of correctly and incorrectly classified samples, the confusion matrix facilitates a nuanced comparison of models beyond mere accuracy, highlighting areas where models excel or fail.

Additionally, the confusion matrix allows for the evaluation of class-specific performance, which is especially valuable when dealing with imbalanced datasets like the FER2013 dataset. For instance, a model may achieve high overall accuracy but perform poorly on certain emotions (e.g., fear or disgust) due to the limited representation of those classes. The confusion matrix makes such discrepancies visible and actionable, guiding the development of more balanced and effective models.

Moreover, the confusion matrix is often regarded as the standard approach in many classification tasks for comparing multiple models due to its ability to provide a granular, interpretable analysis of model performance. It has been extensively used in various domains, from image classification to medical diagnosis, as a robust method for performance comparison [60]. The ability to assess both the correct predictions and the types of errors made by each model makes the confusion matrix particularly valuable in model selection and refinement. Although there are alternative methods of statistical analysis available, such as ROC curves, AUC scores, and cross-validation, the confusion matrix was selected as the most relevant and widely adopted technique for the current task [61]. It directly addresses the core challenges of FER, such as misclassification between similar facial expressions, and provides a straightforward, interpretable summary of model performance [62].

**Figure 3:**
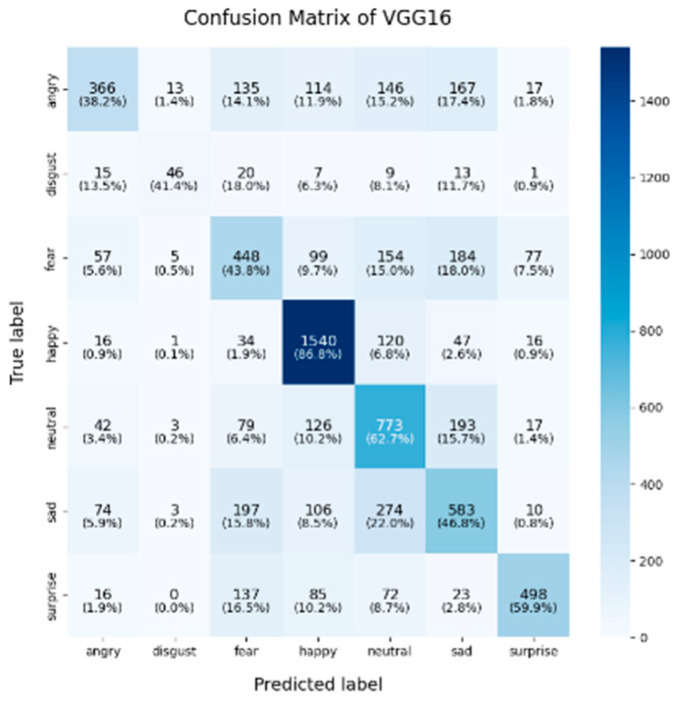
Confusion matrix of VGG16.

**Figure 4:**
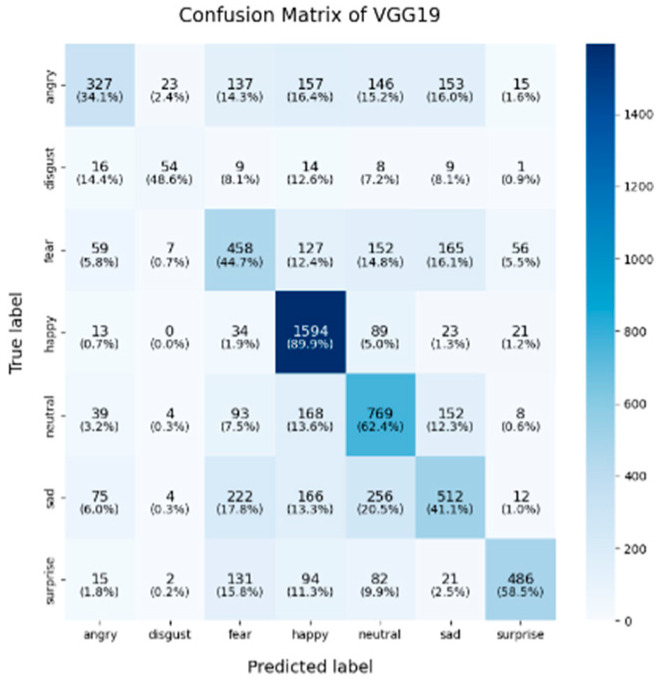
Confusion matrix of VGG19.

**Figure 5:**
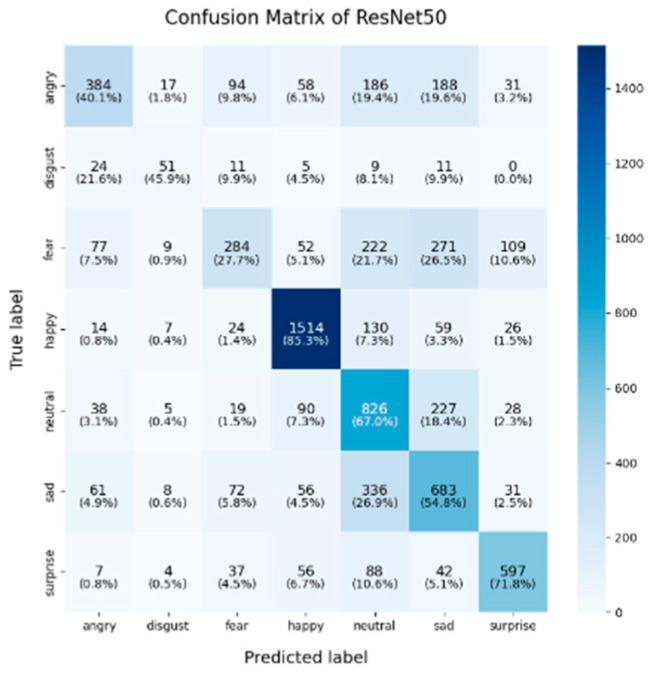
Confusion matrix of Resnet50.

**Figure 6:**
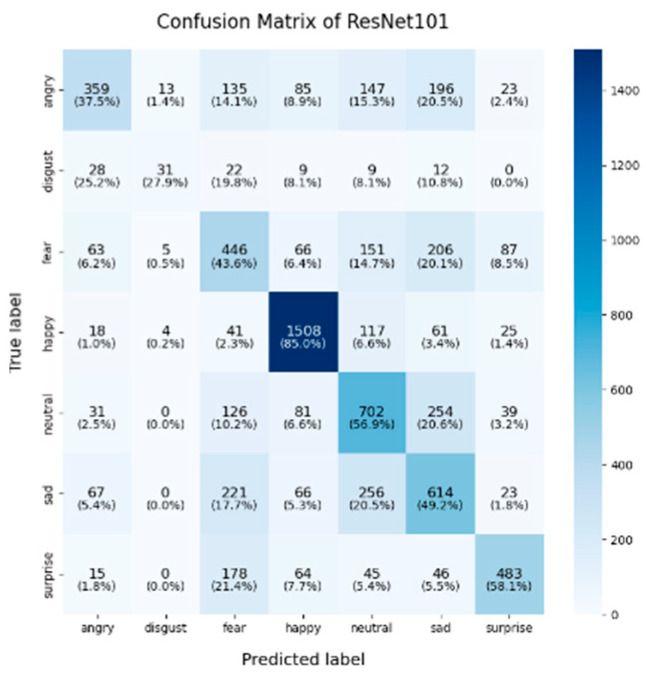
Confusion matrix of Resnet101.

**Figure 7:**
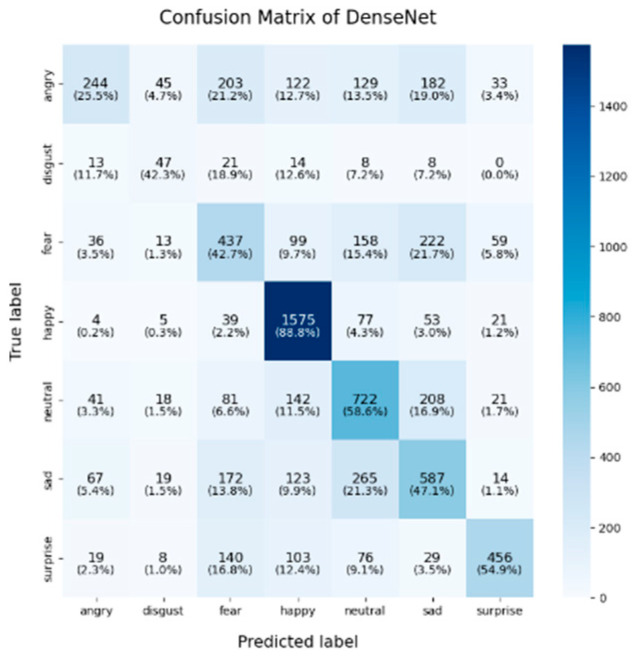
Confusion matrix of DenseNet.

**Figure 8:**
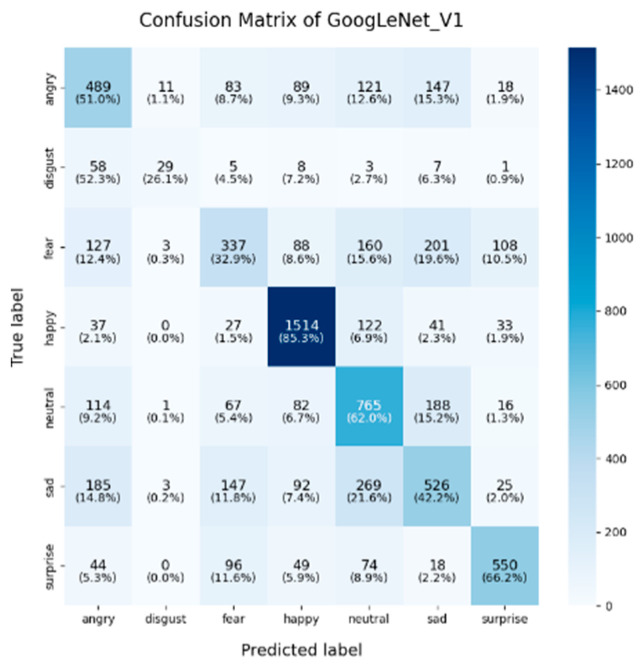
Confusion matrix of GoogLeNet V1.

**Figure 9:**
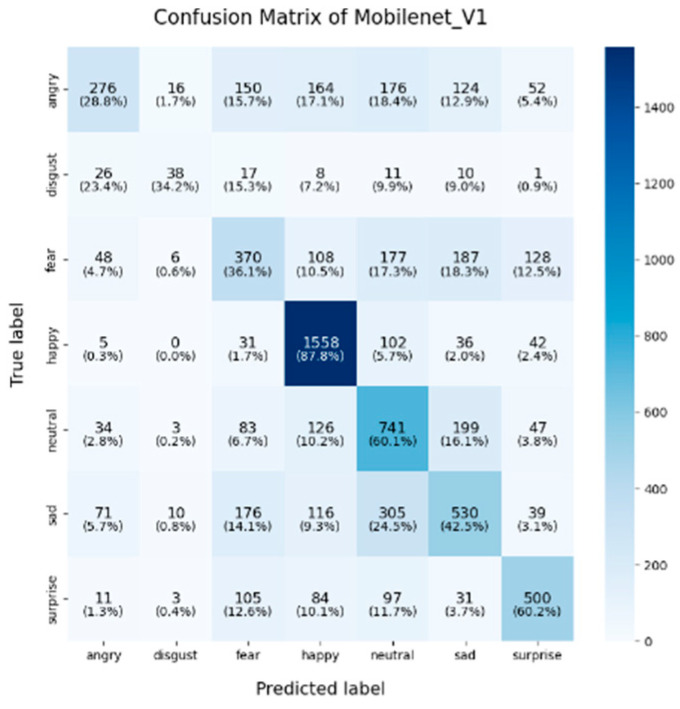
Confusion matrix of MobileNet V1.

**Figure 10:**
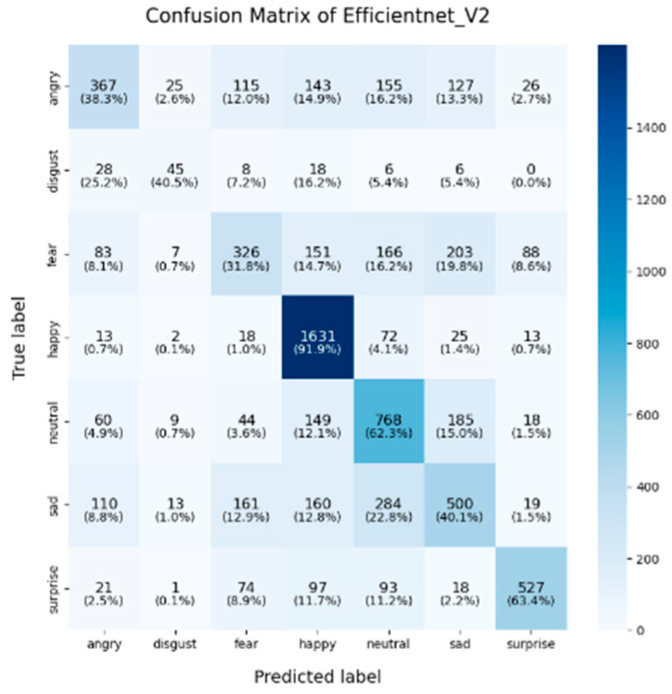
Confusion matrix of EfficientNet V2.

**Figure 11:**
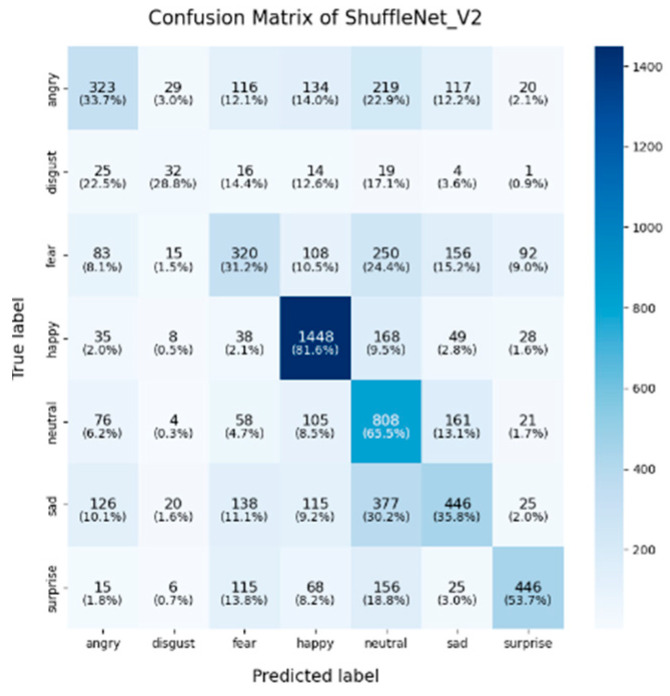
Confusion matrix of ShuffleNet V2.

**Figure 12:**
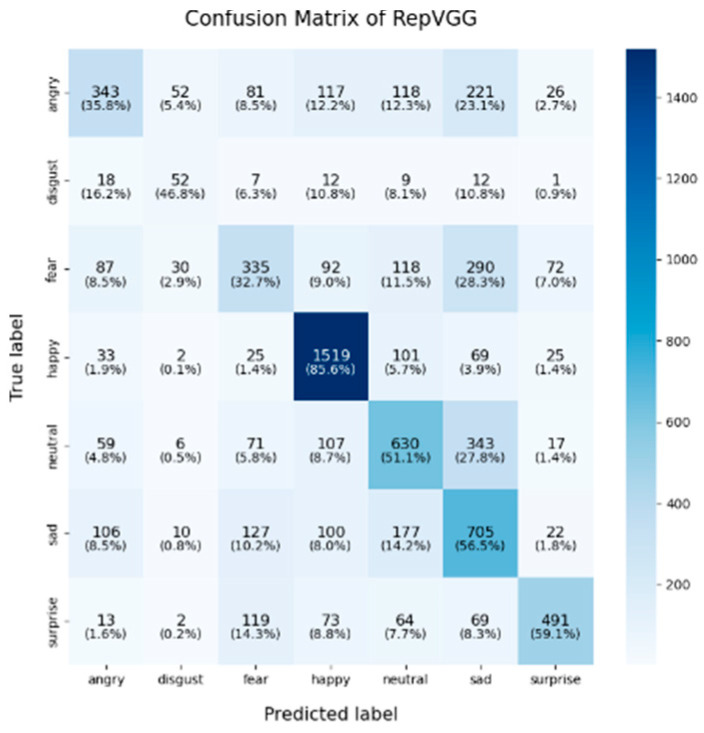
Confusion matrix of RepVGG.

In this study, the confusion matrices illustrating the performance of the selected deep learning models (VGG16, VGG19, ResNet50, ResNet101, DenseNet, GoogLeNet V1, MobileNet V1, EfficientNet V2, ShuffleNet V2 and RepVGG) on the FER2013 dataset are presented in Figure 3, Figure 4, Figure 5, Figure 6, Figure 7, Figure 8, Figure 9, Figure 10, Figure 11 and Figure 12, offering insights into the challenges of facial expression recognition within this experimental context. The convolutional neural networks (CNNs) employed in these models exhibit both shared trends and unique characteristics in their ability to classify facial expressions. A comprehensive analysis of their performance will reveal the common features, distinct aspects, and critical differences that set these models apart from one another.

#### 4.2.1. Uniform Trends in Emotion Recognition Across Models

Across all models, the “happy” label consistently exhibited the highest classification accuracy, with nearly all models surpassing 85%. This trend was particularly evident in models like EfficientNet V2 (91.9%), VGG19 (89.9%), and VGG16 (86.8%). This suggests that these models excel at recognizing features associated with happiness, likely due to the distinct and universally recognizable facial expressions linked to this emotion, such as smiles, which provide clear and distinguishable visual cues. Another contributing factor could be the class imbalance in the FER2013 dataset, where the “happy” label was the most frequent, with nearly 9000 images, potentially leading to this observed characteristic. As for the “neutral” and “surprise” emotions, the models also performed relatively well, with classification accuracies ranging from 50% to 67%. EfficientNet V2, ResNet50, and ShuffleNet V2 excelled in these two categories, with the “neutral” class achieving accuracies of around 65%. This performance suggests that these models can distinguish between the lack of strong facial emotion cues (“neutral”) and the distinct characteristics of “surprise”. However, compared to more expressive emotions like happiness or sadness, “neutral” and “surprise” present additional challenges due to their more subtle facial features.

#### 4.2.2. Model-Specific Strengths and Weaknesses in Emotional Classification

A common challenge across all models in this experiment was the recognition of “fear” and “disgust”, with classification accuracies often below 50%. For instance, DenseNet, ResNet101, and RepVGG struggled with fear classification, achieving accuracies between 32% and 44%, while the “disgust” classification was even more problematic, with EfficientNet V2 and MobileNet V1 yielding accuracies below 50%. This suggests that these emotions may lack distinct, easily recognizable facial features. Additionally, the visual similarities between expressions of “fear” and “surprise”, or between “disgust” and “anger”, may contribute to higher misclassification rates. This is evident in the confusion matrices, where misclassification between emotions such as “angry”, “fear”, and “disgust” is prevalent. The overlapping or similar facial features among these emotions, such as furrowed brows or wide eyes, may lead to higher rates of false positives or false negatives. For example, in DenseNet and GoogLeNet V1, the “fear” label was frequently misclassified as “angry” or “neutral,” while “disgust” was often confused with “angry”.

In this study, EfficientNet V2 and ResNet50 demonstrated exceptional performance across multiple facial expression categories. EfficientNet V2 consistently maintains high accuracy, particularly in the “happy” (91.9%) and “neutral” (62.3%) categories, while ResNet50 excels in the “neutral” (67%) and “surprise” (71.8%) categories. This superior performance can be attributed to the highly optimized architecture of EfficientNet V2, where the compound scaling method effectively balances the network’s depth, width, and resolution. ResNet50, on the other hand, benefited from its residual connections, which help mitigate the vanishing gradient problem in deeper layers, thereby enhancing its ability to differentiate subtle emotional variations. RepVGG exhibited strong and balanced performance across various emotion categories, achieving an accuracy of 85.6% for “happy,” with respectable results in “surprise” (59.1%) and “sadness” (56.5%). Its re-parameterization technique, which facilitates both simplicity and efficiency during inference, likely contributes to its robust performance across different emotions. However, like other models, it struggled in the “fear” and “disgust” categories.

ShuffleNet V2’s mixed performance is noteworthy, as it is designed as a lightweight and efficient model. In this experiment, although ShuffleNet V2 performed well in the “neutral” category (65.5%), it underperformed in the “happy” and “fear” categories, with accuracies of 81.6% and 31.2%, respectively. The architecture’s focus on low computational cost likely limits its ability to capture and distinguish facial micro-expressions, particularly in challenging categories such as “fear” and “disgust”. Finally, despite being based on traditional architectures, both VGG16 and VGG19 remained competitive. VGG19 achieved an accuracy of 89.9% in the “happy” category, and both VGG models show relatively strong performance in the “fear” category, with accuracies of 43.8% (VGG16) and 44.7% (VGG19). However, their performance in the “sadness” and “disgust” categories was outperformed by more modern architectures like EfficientNet V2 and ResNet50, reflecting the limitations of the VGG architecture in capturing more facial micro-emotion variations-

As shown in these 10 figures, the distinguishing characteristics between different models are evident. One of the most notable differences between models such as EfficientNet V2, ResNet50, and VGG architectures lies in the trade-off between model complexity and performance. EfficientNet V2 and ResNet50 leverage more advanced architectural strategies, such as compound scaling and residual connections, which enable them to outperform traditional models like VGG16 and VGG19 in terms of overall accuracy and robustness, particularly in challenging categories such as “neutral” and “surprise”. This underscores the advantage of modern, deeper architectures, which are capable of capturing more subtle features without significantly increasing computational overhead.

#### 4.2.3. Influential Factors in Misclassification and Performance Disparities

In the context of lightweight models, architectures such as ShuffleNet V2 and MobileNet V1 are purposefully designed to enhance computational efficiency, particularly for deployment in mobile and resource-constrained environments. Despite their efficiency, these models tend to exhibit lower overall classification accuracy, particularly in complex emotional categories such as “fear” and “disgust”. Although optimized for speed and reduced computational costs, these architectures demonstrate a limited capacity to capture intricate emotional features when compared to more complex models like ResNet and EfficientNet V2, which ultimately results in suboptimal classification performance. Consequently, in practical scenarios where hardware resources are restricted, the trade-off between model compactness and classification accuracy becomes a crucial factor to consider for deployment.

In conclusion, the comparative analysis of the confusion matrices of the 10 models in this section revealed several consistent patterns, notably the high classification accuracy in the “happy” category and the persistent challenges in recognizing the “fear” and “disgust” emotions. Advanced architectures such as EfficientNet V2 and ResNet50 consistently demonstrate superior performance compared to traditional models like VGG16 and VGG19, highlighting the critical role of modern architectural innovations, including compound scaling and residual connections, in enhancing facial expression recognition. Conversely, lightweight models such as ShuffleNet V2 and MobileNet V1 provide important trade-offs between computational efficiency and classification accuracy, making them well-suited for deployment in resource-constrained environments, although they exhibited diminished performance in more complex emotion categories. These findings emphasize the necessity of selecting models that align with the specific demands of an application, whether the priority lies in maximizing accuracy, optimizing computational efficiency, or achieving a balanced trade-off between the two.

#### 4.2.4. Analysis of Validation Accuracy Trends

**Figure 13:**
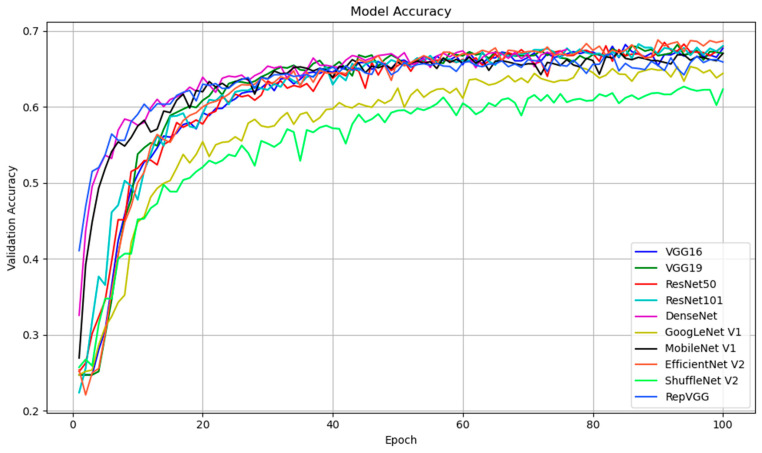
Accuracy for each epoch on models.

Figure 13 illustrates the validation accuracy trend across 100 epochs for 10 existing models: VGG16, VGG19, ResNet50, ResNet101, DenseNet, GoogLeNet V1, MobileNet V1, EfficientNet V2, ShuffleNet V2, and RepVGG.

##### Early-Stage Learning and Convergence Speed

During the early stages of training (up to 20 epochs), the models exhibit significant differences in the rate at which they improve accuracy. Notably, EfficientNet V2, ResNet50, and GoogLeNet V1 demonstrate relatively rapid initial convergence, achieving 55% to 60% accuracy by the 20th epoch. This rapid early improvement suggests that these architectures possess the ability to effectively learn facial expression features, owing to their optimized structural characteristics. EfficientNet V2 employs compound scaling, which balances depth, width, and resolution, allowing the model to capture high-quality facial feature variations in the early stages of training. Similarly, ResNet50 benefits from residual connections, which help mitigate the vanishing gradient problem and accelerate learning by preserving gradients through deeper layers. DenseNet and RepVGG follow closely, with accuracy steadily improving, though their convergence speed is slightly slower than EfficientNet V2 and ResNet50. DenseNet’s dense connections enable parameter reuse and efficient gradient flow, contributing to its smooth learning curve, though it requires more epochs to catch up to the leading models. MobileNet V1 and ShuffleNet V2, designed for efficiency and computational constraints, exhibit slower convergence during the initial training phase, reaching only 45% to 50% accuracy by the 20th epoch. With fewer parameters, these lightweight models require more training epochs to adequately learn facial features. Their slower start can also be attributed to limited depth and feature extraction capacity, reducing their ability to distinguish subtle facial features in the early stages of training.

##### Mid-Stage Stability and Generalization

Between epochs 20 and 60, most models begin to exhibit stabilized performance, characterized by slow but steady improvements in accuracy. EfficientNet V2, ResNet50, GoogLeNet V1, and VGG19 maintain their leading positions, with validation accuracy ranging between 65% and 70% by epoch 60. These models generalize well during this phase, showing minimal signs of overfitting and a relatively consistent upward trend. The superior performance of EfficientNet V2 can be attributed to its compound scaling methodology, which optimally balances network depth, width, and resolution, thereby facilitating efficient feature extraction while minimizing parameter redundancy and computational overhead. ResNet50, with its residual connections, and GoogLeNet V1, utilizing Inception modules, continue to benefit from their architectural designs, facilitating efficient learning in deeper layers. DenseNet and RepVGG also demonstrate strong generalization capabilities, gradually closing the gap with the leading models and converging to an accuracy of approximately 65% by epoch 60.

The progressive improvements in validation accuracy observed in DenseNet and RepVGG can be attributed to DenseNet’s efficient feature reuse through densely connected layers and RepVGG’s re-parameterization strategy, which optimize learning dynamics, despite their relatively slower convergence during the initial training phases. In contrast, MobileNet V1 and ShuffleNet V2 progress more slowly. Although their lightweight network designs are advantageous for mobile and resource-constrained devices, they result in a slower learning process, particularly when dealing with the complexity of facial emotion recognition. By epoch 60, both models achieve accuracy of around 55% to 60%, reflecting the trade-off between computational efficiency and learning capacity.

##### Late-Stage Performance and Convergence

By the end of the 100 training epochs, several models converged to their peak validation accuracy, revealing distinct differences in architectural performance. EfficientNet V2 and ResNet50 outperformed the other models, achieving an accuracy close to 70%. This indicates that these architectures, which balance depth and complexity, are particularly well-suited for the task, demonstrating robust generalization capabilities even after extensive training. Their learning curves remain stable without significant overfitting, suggesting that these models effectively manage the trade-off between bias and variance. In comparison, GoogLeNet V1, DenseNet, and RepVGG maintained accuracy within the 65% to 68% range. These models also exhibited stable convergence, demonstrating that their architectural innovations—Inception modules in GoogLeNet, dense connections in DenseNet, and re-parameterization in RepVGG—contribute to performance stability. The absence of notable fluctuations in accuracy suggests that these models generalize well across the validation set without overfitting the training data. Although VGG16 and VGG19 are more traditional architectures, they still performed well, reaching approximately 65% accuracy by epoch 100. Their slower convergence and slightly lower overall accuracy can be attributed to their reliance on stacked convolutional layers, lacking the advanced innovations and efficiency of more modern networks. MobileNet V1 and ShuffleNet V2, while maintaining a respectable accuracy of around 60%, demonstrated comparatively lower performance than the more complex architectures. Their lightweight design is advantageous for computational efficiency and training time, but it also limits their capacity to capture deep and complex features, which are essential for achieving higher accuracy in facial expression recognition tasks.

In summary, the validation accuracy of these 10 models exhibited significant distinctions in terms of learning efficiency, generalization capability, and architectural strengths. EfficientNet V2 and ResNet50 demonstrated exceptional performance, leveraging advanced architectural designs such as compound scaling and residual connections to achieve high accuracy and stable convergence. Additionally, DenseNet, GoogLeNet V1, and RepVGG also exhibit strong performance, albeit with slower initial learning rates. Finally, while lightweight models such as MobileNet V1 and ShuffleNet V2 are highly efficient, their reduced architectural complexity makes it challenging to reach the same high-performance levels as more complex models. These results highlight the critical importance of selecting the appropriate model architecture based on the trade-off between computational cost and accuracy, particularly in tasks such as facial expression recognition, where the ability to capture subtle features is essential for high accuracy.

## 5. Conclusions

This study presents a comprehensive evaluation of 10 advanced deep learning models—VGG16, VGG19, ResNet50, ResNet101, DenseNet, GoogLeNet V1, MobileNet V1, EfficientNet V2, ShuffleNet V2, and RepVGG—on the challenging task of facial expression recognition using the FER2013 dataset. Through detailed analysis of key performance metrics, including test accuracy, training time, and weight file size, significant differences in learning efficiency, generalization capability, and architectural innovations were observed across these models.

Firstly, EfficientNet V2 and ResNet50 emerged as top performers, achieving high accuracy and stable convergence, owing to their advanced architectural designs, such as compound scaling and residual connections. These mechanisms enhance the models’ ability to capture complex and subtle emotional features, enabling robust generalization even after extensive training. DenseNet, GoogLeNet V1, and RepVGG also demonstrated competitive performance, benefiting from innovations such as dense connectivity, Inception modules, and re-parameterization techniques, although with slower convergence rates in the early stages of training. These models maintained a good balance between computational complexity and feature extraction capability, resulting in reliable overall performance. In contrast, lightweight models such as MobileNet V1 and ShuffleNet V2, while offering exceptional computational efficiency and faster training times, faced challenges in achieving high-precision performance, particularly in complex emotion categories such as “fear” and “disgust”. Their reduced architectural complexity, designed with deployment in mobile and resource-constrained environments in mind, limits their ability to capture intricate facial expressions, highlighting the trade-off between efficiency and performance.

This study underscores the pivotal role of architectural innovation in achieving an optimal balance between classification accuracy and computational efficiency in facial expression recognition tasks. The findings highlight the necessity of a model selection that aligns with the specific operational requirements of the application, whether the emphasis lies in maximizing predictive accuracy, minimizing computational overhead, or achieving a balanced trade-off between these objectives.

Future research will explore the potential of Vision Transformers (ViTs) and other non-CNN architectures, which have exhibited promising performance across various tasks, to further enhance facial expression recognition capabilities. For instance, Ma et al. (2021) [63] proposed the Visual Transformers with Feature Fusion (VTFF) for facial expression recognition, combining CNN-based feature fusion with Vision Transformers (ViT) for modeling global and local attention. The method outperformed existing approaches on multiple in-the-wild datasets, setting new state-of-the-art results on RAF-DB, FERPlus, and AffectNet. Chen et al. (2023) [64] introduced SSF-ViT, a self-supervised vision transformer for few-shot FER, combining self-supervised learning and few-shot learning to improve recognition with fewer labeled samples. The method showed strong performance on FER2013, AffectNet, and RAF-DB, enhancing recognition accuracy for specific expressions. These studies demonstrate the effectiveness of ViT in FER, particularly for handling complex real-world scenarios with limited data. Although ViTs have demonstrated superior accuracy in numerous domains, their substantial computational demands highlight the need for efficient adaptation strategies for deployment in resource-constrained IoT environments. Moreover, advanced approaches, such as hybrid models integrating CNNs with ViTs or lightweight transformer-based architectures, will be investigated to improve both performance and computational efficiency in such systems.

Additionally, future work will also address the limitations of the current dataset by considering other publicly available FER datasets. Although the FER2013 dataset has provided valuable insights, expanding the model’s validation to include more diverse and comprehensive datasets, such as AffectNet, RAF-DB, or the CK+ dataset, will be crucial for evaluating the robustness and generalizability of the model across different environments and populations. Incorporating datasets from various domains, including real-world background, laboratory background, and diverse region populations, will help ensure the broader applicability of facial expression recognition systems.

These insights contribute to the broader advancements in the design and development of deep learning architectures, particularly in fields that demand the recognition of subtle and complex patterns, such as facial expression analysis, where precise feature extraction is paramount to performance. These findings contribute to the academic discourse by emphasizing the pivotal role of model architecture in determining FER performance. Beyond technical metrics, this study emphasized the practical implications of FER across diverse domains such as big data, healthcare, education, and security, where these technologies can enhance human-computer interaction, refine customer analytics, and bolster safety protocols. Looking ahead, future research endeavors should concentrate on refining model architectures to improve robustness and generalizability across varied datasets and real-world conditions. Addressing challenges such as data imbalance and environmental variability will be crucial for advancing the practical applications of FER technologies.

## Figures and Tables

**Table 1:** FER 2013 Dataset Information.

Facial Expression	Number of Images	Training Set Instances	Test Set Instances
Angry	4983	3995	958
Happy	8989	7215	1774
Fear	5121	4097	1024
Disgust	547	436	111
Sad	6077	4830	1247
Surprise	4002	3171	831
Neutral	6198	4965	1233

**Table 2:** Training Parameters.

Learning Rate	Batch Size	Epochs	Optimizer
0.001	64	100	Adam

**Table 3:** Deep Learning Model Comparison.

Model	Number of Conv Layers	Number of FC Layers	Activation Function	BN ^1^	Key Feature
VGG16	13	3	ReLU	Yes	Deep architecture, uniform architecture with small filters, good generalization.
VGG19	16	3	ReLU	Yes	Deep architecture with large kernel sizes captures more complex features.
ResNet50	49	1	ReLU	Yes	Residual learning framework enables the training of intense networks with improved stability.
ResNet101	100	1	ReLU	Yes	Residual learning mitigates the challenges associated with vanishing and exploding gradients.
DenseNet	118	1	ReLU	Yes	Each layer receives feature maps from all previous layers in a dense connectivity pattern.
GoogLeNet V1	22	1	ReLU	Yes	Inception module is efficient at capturing features at different spatial scales.
MobileNet V1	27	1	ReLU	Yes	Depthwise separable convolution efficiently replaces traditional convolution operations.
EfficientNet V2	53	1	ReLU	Yes	The introduction of the Fused-MBConv architecture and progressive learning strategies.
ShuffleNet V2	24	1	ReLU	Yes	Efficient computation is achieved through the rearrangement of channels.
RepVGG	22 (5 stage)	1	ReLU	Yes	Multi-branch during training, single branch during inference.

^1^ BN represents Batch Normalization.

**Table 4:** Model Evaluation Metric.

Model	Test Accuracy (%)	Training Time (S/Epoch) ^1^	Weight File Size (MB)
VGG16	67.7	79.8	512
VGG19	67.1	80.6	512
ResNet50	68.1	44.4	81.3
ResNet101	68.1	44.7	158
DenseNet	67.6	54.3	27.1
GoogLeNet V1	64.4	27.8	39.4
MobileNet V1	67.1	30.8	12.3
EfficientNet V2	**68.7**	46.2	77.8
ShuffleNet V2	62.3	**20.6**	**1.47**
RepVGG	66.1	21.6	10.7

^1^ S/Epoch represented the number of seconds required for each round of model training.

## Data Availability

Data are contained within the article.
